# Incidence and prevalence of major epilepsy-associated brain lesions

**DOI:** 10.1016/j.ebr.2022.100527

**Published:** 2022-02-11

**Authors:** Javier A. López-Rivera, Victoria Smuk, Costin Leu, Gaelle Nasr, Deborah Vegh, Arthur Stefanski, Eduardo Pérez-Palma, Robyn Busch, Lara Jehi, Imad Najm, Ingmar Blümcke, Dennis Lal

**Affiliations:** a Department of Molecular Medicine, Cleveland Clinic Lerner College of Medicine, Case Western Reserve University, USA; b Genomic Medicine Institute, Lerner Research Institute, Cleveland Clinic, USA; c Epilepsy Center, Neurological Institute, Cleveland Clinic, Cleveland, USA; d Stanley Center for Psychiatric Research, Broad Institute of MIT and Harvard, Cambridge, MA, USA; e Universidad del Desarrollo, Centro de Genética y Genómica, Facultad de Medicina Clínica Alemana. Santiago, Chile; f Department of Psychiatry and Psychology, Neurological Institute, Cleveland Clinic, Cleveland, OH, USA; g Department of Neurology, Neurological Institute, Cleveland Clinic, Cleveland, OH, USA; h Department of Neuropathology, University Hospital Erlangen, Erlangen, Germany; i Analytic and Translational Genetics Unit, Massachusetts General Hospital, Boston, MA, USA; j Cologne Center for Genomics, University of Cologne, Cologne, NRW, Germany

**Keywords:** Epilepsy, Epilepsy surgery, Malformation of cortical development, Hippocampal sclerosis, Low-grade Epilepsy-associated tumor

## Abstract

Epilepsy surgery is an effective treatment option for drug-resistant focal epilepsy patients with associated structural brain lesions. However, little epidemiological data are available regarding the number of patients with these lesions. We reviewed data regarding (1) the prevalence and incidence of epilepsy; (2) the proportion of epilepsy patients with focal epilepsy, drug-resistant epilepsy, and drug-resistant focal epilepsies; and (3) the number of epilepsy presurgical evaluations and surgical resections. We also assessed the relative proportion of brain lesions using post-surgical histopathological findings from 541 surgical patients from the Cleveland Clinic and 9,523 patients from a European multi-center cohort. Data were combined to generate surgical candidate incidence and prevalence estimates and the first lesion-specific estimates for hippocampal sclerosis (HS), low-grade epilepsy-associated brain tumors (LEAT), malformations of cortical development (MCD), glial scars, vascular malformations, and encephalitis. The most frequently diagnosed brain lesions were HS (incidence = 2.32 ± 0.26 in 100,000, prevalence = 19.40 ± 2.16 in 100,000) for adults and MCD (incidence = 1.15 ± 0.34 in 100,000, prevalence = 6.52 ± 1.89 in 100,000) for children. Our estimates can guide patient advocacy groups, clinicians, researchers, policymakers in education, development of health care strategy, resource allocation, and reimbursement schedules.

## Highlights

• We combined multiple datasets to estimate the epidemiology of epileptogenic brain lesions.
• We provide brain lesion-specific prevalence and incidence estimates in adults and children.
• The estimates may help infer the number of epilepsy surgery candidates in the general population.

## Introduction

It has been estimated that approximately a third of people with epilepsy can be classified as drug-resistant and that this proportion could be higher for people with focal epilepsies [1]. Surgery is especially effective for drug-resistant focal epilepsy patients that have associated structural brain lesions such as hippocampal sclerosis (HS), low-grade developmental and epilepsy-associated brain tumors (LEAT), and malformations of cortical development (MCD) such as focal cortical dysplasia (FCD) [2], [3].

Although the prevalence and incidence of epilepsy have been well-established by multiple epidemiological studies in both adults and children, no population-based studies on the frequency of epilepsy-associated brain lesions in the general population have ever been performed. As such, epidemiological estimates for the incidence and prevalence of these lesions in the pediatric and adult general population remain unknown. However, the proportions of epilepsy-associated brain lesions surgically resected from drug-resistant epilepsy patients–including HS, FCD, and LEAT–have been established through histopathological studies [2], [4].

In order to improve diagnostic and surgical treatment settings, the frequency of surgically treatable epilepsy and epilepsy-associated brain lesions in the general population must be better understood. Such data are required to optimize resource allocation for health care services, including the training of specialists, the types of hospital and support services provided, and the implementation of public health programs. Herein, we combined epidemiological data from the literature with histopathological findings from surgical patients from the Cleveland Clinic and a European multicenter cohort to generate incidence and prevalence estimates of resective surgical candidates. We also provide the first lesion-specific estimates for hippocampal sclerosis (HS), low-grade epilepsy-associated brain tumors (LEAT), malformations of cortical development (MCD), glial scars, vascular malformations, and encephalitis for both adults and children in the general population based on data from the last decades.

## Methods

### Approach to estimate surgical burden and brain lesion prevalence and incidence

In order to estimate the prevalence and incidence of surgically resectable epilepsy among adults and children, our overall approach was to make use of existing and related epidemiological data to calculate estimates. We first performed a systematic review to identify studies with data related to the frequency of epilepsy, drug-resistant focal epilepsy, and epilepsy surgery. Then, we progressively combined available epidemiological data points into all possible sequences starting from the overall prevalence or incidence of epilepsy and ending at the prevalence or incidence of epilepsy surgery candidates (see Fig. 2). For example, we estimated the prevalence of surgical candidates by combining the prevalence of epilepsy with the rate of focal epilepsy among epilepsies, the rate of drug-resistant epilepsy among focal epilepsies, and the rate of epilepsy surgery among patients with drug-resistant focal epilepsy. The estimated prevalence or incidence of surgical candidates was then combined with histopathological classification results from patients who underwent resective epilepsy surgery to calculate the individual prevalence and incidence of common types of epilepsy-associated brain lesions.

### Study identification by systematic review

In order to identify studies with data related to the frequency of epilepsy surgery, we conducted a systematic literature review of the literature using the PubMed database on May 2020 according to the Preferred Reporting Items for Systematic Reviews and Meta-Analyses (PRISMA) guidelines [5].

### Study identification of epilepsy subtypes for both adults and children

**Fig. 1:**
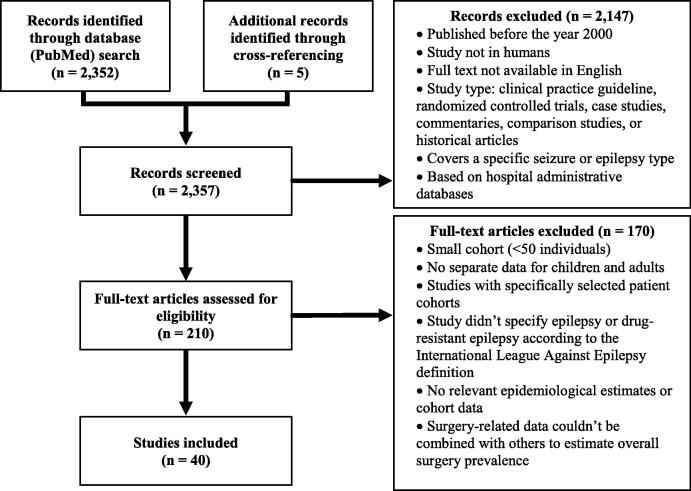
Flow diagram of the comprehensive literature review. n = number of studies. Exclusion criteria are included for both the initial title and abstract screen, as well as the full-text review.

Using multiple search queries (Table 1), we identified studies with epidemiological data associated with epilepsy surgery including (1) the prevalence and incidence of epilepsy; (2) the proportion of epilepsy patients with focal epilepsy, drug-resistant epilepsy, and drug-resistant focal epilepsies; (3) rates of presurgical evaluation and subsequent selection for surgery in epilepsy patients; and (4) published reports specifying the frequency of various epilepsy-associated brain lesions in surgically-resected brain tissue (Table 1). The search was limited to studies published between January 1, 2000 and May 28, 2020. For articles involving drug-resistant epilepsy, we only considered those which adhered to the definition of drug-resistant epilepsy given by the International League Against Epilepsy [6]. Two independent reviewers manually screened the resulting articles at the title, abstract, and full text level to eliminate reports that met our exclusion criteria (Fig. 1). We extracted data from full text articles that met inclusion criteria and cross-referenced the included studies to identify additional studies that met our selection criteria (Table 1; Appendix S1).

**Table 1:** PubMed search queries utilized for literature review.

Epidemiological Estimate	PubMed Search Query
Prevalence of epilepsy	Epilepsy AND Prevalence AND Epidemiological OR Meta-Analysis
Prevalence of epilepsy subtypes	Epilepsy AND Focal OR Drug Resistant OR Intractable OR Refractory AND Prevalence
Focal epilepsy studies	Epilepsy AND Focal AND Cohort OR Frequency OR Population AND Retrospective(ly) OR Prospective(ly)
Drug-resistant epilepsy studies	Epilepsy AND Drug Resistant OR Intractable OR Refractory AND Cohort OR Frequency OR Population AND Retrospective(ly) OR Prospective(ly) OR (a)etiology
Epilepsy surgery and surgical evaluation studies	Epilepsy AND Surgery AND Evaluation(s) AND Prevalence OR Cohort(s) OR Candidacy AND Focal OR Drug Resistant OR Intractable OR Refractory

**Fig. 2:**
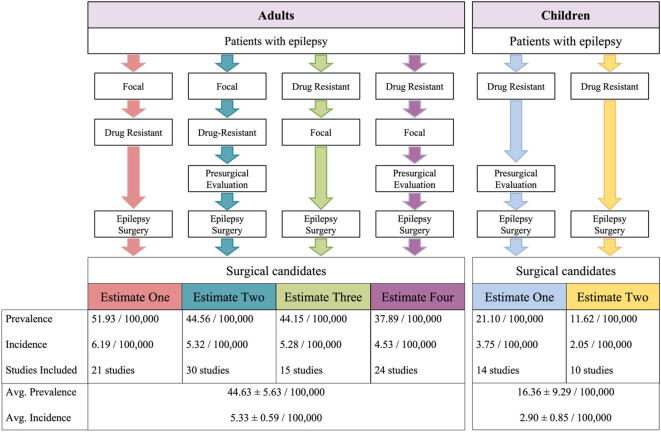
Estimation of annual period prevalence and incidence in the general population of candidates for epilepsy surgery based on reported epidemiological estimates. Each box represents a literature-reported estimate or calculated pooled estimate (see Methods for details and Table 2 for specific values used for calculations). Surgical candidate prevalence and incidence estimates were calculated by sequentially combining these pooled estimates. For example, to generate prevalence estimate one for adults, the estimated prevalence was calculated by combining the annual period prevalence of epilepsy with the rate of focal epilepsy among epilepsies, the rate of drug-resistant epilepsy among focal epilepsies, and the rate of epilepsy surgery among patients with drug-resistant focal epilepsy.

### Study selection and generation of epilepsy-related epidemiological data points

We stratified studies based on the type of reported epidemiological data points, whether the studies were meta-analyses or cohort studies, and the reported age groups (0–17 years of age for children, 18 years of age or older for adults). For reports regarding the presurgical evaluation of patients for epilepsy surgery, we considered the fraction of patients which were selected for surgery after a multi-disciplinary pre-surgical evaluation and not just the fraction of evaluated patients who ultimately underwent surgery. If one or more meta-analyses for an epidemiological data point was available, only the largest and most recent meta-analysis was considered. For epilepsy-related epidemiological data points with no available meta-analyses and more than one independent report that met our study criteria, a weighted mean was calculated using the R package “meta” [7]. Weighted means were calculated using a random effects model to account for variation between studies. For epidemiological data points with only a single report that met our study criteria, only the singular reported data point was considered. We also conducted sensitivity analysis by examining the effect of (1) no stratification based on age, (2) more permissive article inclusion criteria and (3) using a fixed effects model to calculate weighted averages.

### Collection of surgical evaluation and surgery rate data from the Cleveland Clinic Epilepsy Center (2018–2019)

In addition to published data (Appendix S1), we acquired data from the Cleveland Clinic Epilepsy Center Outcomes Registry (CCF ECOR) database regarding the number of patients who underwent pre-surgical evaluation, the proportion of these patients selected for surgery, and the number of patients who ultimately underwent surgical treatment during the years 2018 and 2019 as data were only available for this time period (Table 1). The data from the CCF ECOR was considered when calculating a weighted mean for the proportion of patients considered surgical candidates after presurgical evaluation.

### Collection of histopathological outcomes data from the Cleveland Clinic Epilepsy Center (2010–2018)

In addition to published data [2], we acquired data from the CCF ECOR database regarding 541 patients with surgically treated epilepsy who underwent surgery during the period from 2010 to 2018 in our center. Data were only available for this time period. We combined this data with previously published data from the European Epilepsy Brain Bank consortium (EEBB) to then calculate both annual period prevalence and incidence estimates for the most common surgically treatable epilepsy-associated brain lesions by combining surgical candidate estimates with post-surgical histopathological findings (Table 4). Histopathological review of resected brain tissue was performed and interpreted by board-certified clinical neuropathologists at the Cleveland Clinic Foundation in all patients and a detailed re-review by one of the co-authors (IB). Before surgery, all patients underwent an extensive evaluation that was followed by a discussion at a multidisciplinary patient management conference where a surgical strategy was developed. The Outcomes Registry is approved by the Institutional Review Board of the Cleveland Clinic. Written informed consent for the use of histopathological data was obtained from patients or their representative.

Histopathological diagnosis was based on light microscopic inspection of formalin-fixed paraffin-embedded tissue blocks stained with hematoxylin and eosin or additional histochemical and immunohistological stainings when indicated [8]. Hippocampal sclerosis was defined histopathologically by segmental neuronal cell loss in anatomical sectors of the cornu ammonis of the hippocampus, as specified in the consensus classification of the ILAE [9]. Brain tumors were classified according to the WHO classification of tumors of the central nervous system [10], [11]. Focal cortical dysplasia was defined according to the consensus classification system of the ILAE [12]. Vascular malformations included cavernoma and meningeal angiomatosis, excluding ischemic or hemorrhagic stroke. Glial scars included traumatic brain injury and perinatal infarcts, excluding postsurgical scarring. Encephalitis included Rasmussen, limbic, or any other focal infection, excluding any inflammatory response to intracerebral neurophysiology recordings.

## Results

### Collection of epidemiological data points related to resective epilepsy surgery

Our PubMed literature review identified a total of 2352 unique articles (see Methods, Fig. 1). An initial title and abstract screen yielded 210 full-text articles for further consideration. Of these 210 abstracts, 35 articles met all criteria for final inclusion in our analysis (see Methods). We also considered five additional studies which were identified through cross-referencing articles which met our inclusion criteria, for a total of 40 articles included in our analysis (Fig. 1).

### Collection of data points for adults and children

Of the 40 articles, nine only reported prevalence estimates or rates related to pediatric epilepsy, 28 were only related to adult epilepsy, and three provided separate estimates for both.

### Collection of data points for epilepsies

Our literature review identified epidemiological data associated with epilepsy surgery, such as the annual prevalence or incidence of epilepsy, the prevalence or rate of focal or drug-resistant epilepsy, and rates of pre-surgical evaluation and epilepsy surgery (Table 2). There were ten instances where multiple sources were available for a single data point. Since the overarching goal of this study was to identify data points for incidence and prevalence calculations, we calculated weighted averages for these data points from the overlapping sources (see Methods).

**Table 2:** Studies included and calculated pooled epidemiological estimates.

Study	PMID	Study Type	Adults			Children		
			Events	Total	Proportion [CI]	Events	Total	Proportion [CI]
**Annual period prevalence of active epilepsy**								
Fiest et al., [15]	27986877	Meta-Analysis		22 studies	543/100,000		22 studies	480/100,000

**Cumulative incidence of epilepsy**								
Fiest et al., [15]	27986877	Meta-Analysis		3 studies	64.81/100,000		5 studies	85.29/100,000

**Proportion of epilepsy patients with DRE**								
Aaberg et al., [27]	29789444	Cohort	-	-	-	178	600	0.3
Berg et al., [60]	16685695	Cohort	-	-	-	142	613	0.23
Boonluksiri et al., 2015	26819940	Cohort	-	-	-	129	308	0.42
Gandy et al., [43]	23201610	Cohort	61	130	0.47	-	-	-
Geerts et al., [48]	22417003	Cohort	-	-	-	50	413	0.12
Giussani et al., [33]	26731716	Cohort	83	584	0.14	24	100	0.24
Hui et al., [57]	17628339	Cohort	103	260	0.4	-	-	-
Kong et al., [38]	24910376	Cohort	120	557	0.22	-	-	-
Nickels et al., [44]	22989286	Cohort	-	-	-	134	467	0.29
Picot et al., [56]	18363709	Cohort	81	360	0.22	-	-	-
Ramos-Liziana et al., [54]	19328019	Cohort	-	-	-	30	343	0.09
Sills et al., [62]	15857428	Cohort	230	400	0.57	-	-	-
Tellez-Zenteno et al., [39]	24828683	Cohort	82	250	0.33	-	-	-
**Pooled Estimate**				**7 studies**	**0.32** **[0.21; 0.46]**		**7 studies**	**0.22** **[0.16; 0.31]**

**Proportion of epilepsy patients with FE**								
Bosak et al., 2019	31077939	Cohort	458	653	0.7	-	-	-
Chen et al., [28]	28475999	Cohort	2911	4116	0.71	-	-	-
El-Tallawy et al., [45]	27257380	Cohort	113	198	0.57	-	-	-
Fong et al., [66]	12904612	Cohort	408	736	0.55	-	-	-
Gandy et al., [43]	23201610	Cohort	101	130	0.78	-	-	-
Garcia-Martin et al., [47]	22749918	Cohort	389	515	0.76	-	-	-
Guekht et al., [30]	21035312	Cohort	1430	1753	0.82	-	-	-
Guekht et al., [51]	28142100	Cohort	818	1351	0.61	-	-	-
Hamer et al., [58]	17201718	Cohort	77	101	0.76	-	-	-
Hunter et al., [46]	22883631	Cohort	208	291	0.71	-	-	-
Nguyen et al., [42]	23419568	Cohort	843	1051	0.8	-	-	-
Oun et al., [67]	12536056	Cohort	294	396	0.74	-	-	-
Picot et al., [56]	18363709	Cohort	229	360	0.64	-	-	-
Sills et al., [62]	15857428	Cohort	270	400	0.68	-	-	-
Subramaniam et al., 2020	32094071	Cohort	116	211	0.55	-	-	-
Tellez-Zenteno et al., [39]	24828683	Cohort	142	250	0.57	-	-	-
**Pooled Estimate**				**16 studies**	**0.69** **[0.64; 0.73]**			

**Proportion of FE patients with DRE**								
Garcia et al., 2014	25616468	Cohort	248	515	0.48	-	-	-
Gilioli et al., [49]	22360822	Cohort	453	1155	0.39	-	-	-
Tellez-Zenteno et al., [39]	24828683	Cohort	52	142	0.37	-	-	-
**Pooled Estimate**				**3 studies**	**0.42** **[0.35; 0.49]**			

**Proportion of DRE patients with FE**								
Alexandre et al., [52]	20132292	Cohort	782	933	0.84	-	-	-
Choi et al., [32]	27265407	Cohort	282	403	0.7	-	-	-
Conte et al., [26]	30308426	Cohort	512	640	0.8	-	-	-
Gandy et al., [43]	23201610	Cohort	56	61	0.92	-	-	-
Kong et al., [38]	24910376	Cohort	66	120	0.55	-	-	-
Picot et al., [56]	18363709	Cohort	61	81	0.75	-	-	-
**Pooled Estimate**				**6 studies**	**0.77** **[0.68; 0.84]**			

**Proportion of patients with DRE who underwent surgery**								
Berg et al., [53]	19638447	Cohort	-	-	-	11	132	0.08
Lim et al., [41]	24192043	Cohort	-	-	-	53	463	0.11
**Pooled Estimate**							**2 studies**	**0.11** **[0.08; 0.14]**

**Proportion of patients with focal DRE who underwent surgery**								
Fois et al., 2016	25935890	Cohort	204	612	0.33	-	-	-

**Proportion of patients with DRE who underwent presurgical evaluation**								
Berg et al., [53]	19638447	Cohort	-	-	-	54	132	0.41
Lim et al., [41]	24192043	Cohort	-	-	-	160	463	0.35
**Pooled Estimate**							**2 studies**	**0.37** **[0.31; 0.43]**

**Proportion of patients with focal DRE who underwent presurgical evaluation**								
Dugan et al., [29]	28378422	Cohort	200	407	0.49	-	-	-
Fois et al., 2016	25935890	Cohort	306	612	0.5	-	-	-
Roberts et al., [37]	25107882	Cohort	42	107	0.39	-	-	-
**Pooled Estimate**				**3 studies**	**0.48** **[0.43; 0.52]**			

**Proportion of patients who underwent surgery after presurgical evaluation**								
Berg et al., [64]	14636351	Cohort	368	522	0.7	-	-	-
Cleveland Clinic, 2018 – 2019	n/a	Cohort	225	388	0.58	217	271	0.8
Cloppenborg et al., [22]	30577071	Cohort	1357	1916	0.71	751	1300	0.58
Conte et al., [26]	30308426	Cohort	109	249	0.44	-	-	-
Dugan et al., [29]	28378422	Cohort	113	200	0.56	-	-	-
Fois et al., 2016	25935890	Cohort	204	306	0.67	-	-	-
Haque et al., [35]	26092414	Cohort	-	-	-	51	131	0.39
Lim et al., [41]	24192043	Cohort	-	-	-	53	160	0.33
Picot et al., [31]	27595433	Cohort	119	289	0.41	-	-	-

**Pooled Estimate**				**7 studies**	**0.59** **[0.49; 0.68]**		**4 studies**	**0.54** **[0.35; 0.71]**

CI = confidence intervals; DRE = drug-resistant epilepsy; FE = focal epilepsy.

### Estimating the prevalence and incidence of resective epilepsy surgery candidates by combining literature-derived data points

Evidence-based estimates for the incidence and prevalence of surgically treatable epilepsy in the general population have yet to be established. In order to estimate the annual period prevalence and incidence of resective epilepsy surgery candidates in both the adult and pediatric general population, we combined data points from reported epidemiological data and calculated literature-derived weighted averages (Table 2). We made use of different combinations of these data points to derive multiple estimates for both prevalence and incidence (Fig. 2). Four surgical candidate prevalence and incidence estimates were derived for adults from different combinations and two were derived for children. For adults, based on an estimated annual period prevalence of active epilepsy of 543 in 100,000 and an incidence of epilepsy of 64.81 in 100,000 adults, we estimated an average annual period prevalence of 44.63 ± 5.63 and an average incidence of 5.33 ± 0.59 surgical candidates in 100,000 adults. Based on these estimates, 8.2% of adults with epilepsy would qualify as surgical candidates. For children, based on an estimated annual period prevalence of active epilepsy of 480 in 100,000 and an incidence of epilepsy of 85.29 in 100,000 children, we estimated an average annual period prevalence of 16.36 ± 9.29 and an average incidence of 2.90 ± 0.85 surgical candidates in 100,000 children. Based on these estimates, 3.4% of children with epilepsy would qualify as surgical candidates. Performing the same analysis without age-based study stratification or age-based study exclusion resulted in estimates similar to those calculated for adults (Fig. S1 and Table S1).

We also combined literature-derived weighted means (Table 2) to estimate an average prevalence of focal drug-resistant epilepsy among adults of 145.58 in 100,000 and a prevalence of drug-resistant epilepsy among children of 105.6 in 100,000. In order to estimate the proportion of patients with drug-resistant epilepsy which would qualify as candidates for resective epilepsy surgery, we took the proportion between these estimates and the surgical candidate average prevalence estimates (Fig. 2). From this, we estimate that 30.66% of adults with focal drug-resistant epilepsy and 15.5% of children with drug-resistant epilepsy would qualify as candidates for epilepsy surgery.

Due to high heterogeneity between the studies included in the pooled estimates (Figs. S2-S11), we made use of a fixed effects model when calculating our estimates. Regardless, sensitivity analysis with a random effects model did not significantly alter the calculated estimates (Table S2). Additionally, to further evaluate potential study selection bias due to our exclusion criteria (Fig. 1), we performed an additional sensitivity analysis with less strict article inclusion criteria. For this analysis, we did not exclude (i) studies based on hospital administrative databases and (ii) studies that reported on drug-resistant epilepsy but did not meet or specify the standard definition of drug-resistant epilepsy according to the International League Against Epilepsy (*N* = 14, Table S1). Including these additional 14 data points resulted in incidence and prevalence estimates similar to our main analysis (Table S2).

### Estimating the prevalence and incidence of surgically resectable epilepsy-associated brain lesions

To date, no study has reported on the population frequency of common focal epilepsy-associated lesions such as hippocampal sclerosis and MCD. We first generated weighted average proportions for each major epilepsy-associated brain lesion by combining data from the CCF ECOR database regarding 541 patients who underwent surgical resection between 2010 and 2018 (408 adults and 133 children) and published data from an independent multi-center European series of 9,523 patients (6,900 adults and 2,623 children) from the European Epilepsy Brain Bank consortium (EEBB) who underwent surgical resection between 1990 and 2014 [2] (Table 3). We then calculated both annual period prevalence and incidence estimates for the most common surgically treatable epilepsy-associated brain lesions by combining the adult and pediatric surgical candidate estimates with post-surgical histopathological findings from adults and children (Table 4). Using the combined histopathological findings data, hippocampal sclerosis was the most common surgically treatable brain lesion among adults and MCDs were the most common surgically treatable brain lesions among pediatric patients. Among the MCDs, FCD II was the most prevalent.

**Table 3:** Proportion of major epilepsy-associated brain lesions among adults and children undergoing epilepsy surgery.

Lesion type	CCF ECOR 2010–2018 (%)	EEBB 1990–2014 (%)	Weighted Average Proportion (%) [95% CI]
**Adults**	**408**	**6,900**	
Hippocampal Sclerosis	106 (25.98%)	3,070 (44.49%)	43.46% [42.33; 44.60]
MCD	131 (32.11%)	856 (12.41%)	13.51% [12.74; 14.31]
FCD I	6 (1.47%)	101 (1.46%)	1.46% [1.21; 1.77]
FCD II	55 (13.48%)	412 (5.97%)	6.39% [5.85; 6.97]
FCD (NOS)	3 (0.74%)	118 (1.71%)	1.66% [1.39; 1.98]
Other MCD	67 (16.42%)	225 (3.26%)	4% [3.57; 4.47]
LEAT	35 (8.58%)	1,530 (22.17%)	21.41% [20.49; 22.37]
Glial scar	36 (8.82%)	311 (4.51%)	4.75% [4.28; 5.26]
Vascular malformation	17 (4.17%)	497 (7.20%)	7.03% [6.47; 7.64]
Encephalitis	5 (1.23%)	59 (0.86%)	0.88% [0.69; 1.12]

**Children**	**133**	**2,623**	
Hippocampal Sclerosis	15 (11.28%)	394 (15.02%)	14.84% [13.56; 16.22]
MCD	66 (49.62%)	1032 (39.34%)	39.84% [38.03; 41.68]
FCD I	2 (1.50%)	167 (6.37%)	6.13% [5.3; 7.09]
FCD II	41 (30.83%)	447 (17.04%)	17.71% [16.33; 19.18]
FCD (NOS)	1 (0.75%)	88 (3.35%)	3.23% [2.63; 3.96]
Other MCD	22 (16.54%)	333 (12.70%)	12.88% [11.68; 14.18]
LEAT	19 (14.29%)	714 (27.22%)	26.6% [24.98; 28.28]
Glial scar	14 (10.53%)	153 (5.83%)	6.06% [5.23; 7.01]
Vascular malformation	2 (1.50%)	84 (3.20%)	3.12% [2.53; 3.84]
Encephalitis	5 (3.76%)	86 (3.28%)	3.3% [2.7; 4.04]

CCF ECOR = Cleveland Clinic Epilepsy Center Outcomes Registry database, EEBB = European Epilepsy Brain Bank, MCD = Malformation of cortical development, FCD I = Focal cortical dysplasia type I, FCDII = Focal cortical dysplasia type II, FCD (NOS) = Focal cortical dysplasia (not otherwise specified), LEAT = Low-grade developmental and epilepsy-associated brain tumors.

**Table 4:** Estimated annual period prevalence and incidence of epilepsy-associated brain lesions in adults and children in the general population.

	General population period prevalence in 100,000					General population incidence in 100,000				
Lesion type	Est. 1	Est. 2	Est. 3	Est. 4	Average ± SD	Est. 1	Est. 2	Est. 3	Est. 4	Average ± SD
**Adults**										
Hippocampal Sclerosis	22.57	19.37	19.19	16.47	**19.40 ± 2.16**	2.69	2.30	2.30	1.97	**2.32 ± 0.26**
MCD	7.02	6.02	5.96	5.12	**6.03 ± 0.67**	0.84	0.71	0.71	0.62	**0.72 ± 0.08**
FCD I	0.76	0.65	0.64	0.55	**0.65 ± 0.07**	0.09	0.07	0.07	0.07	**0.08 ± 0.01**
FCD II	3.32	2.85	2.82	2.42	**2.85 ± 0.32**	0.39	0.34	0.34	0.28	**0.34 ± 0.04**
FCD (NOS)	0.86	0.74	0.73	0.63	**0.74 ± 0.08**	0.09	0.09	0.09	0.07	**0.09 ± 0.01**
Other MCD	2.08	1.78	1.77	1.52	**1.79 ± 0.20**	0.24	0.21	0.21	0.19	**0.21 ± 0.02**
LEAT	11.12	9.54	9.45	8.11	**9.56 ± 1.07**	1.33	1.14	1.12	0.97	**1.14 ± 0.13**
Glial scar	2.47	2.12	2.10	1.80	**2.12 ± 0.24**	0.30	0.24	0.24	0.21	**0.25 ± 0.03**
Vascular malformation	3.65	3.13	3.10	2.66	**3.14 ± 0.35**	0.43	0.37	0.37	0.32	**0.37 ± 0.04**
Encephalitis	0.46	0.39	0.39	0.33	**0.39 ± 0.05**	0.06	0.04	0.04	0.04	**0.04 ± 0.01**

**Children**										
Hippocampal Sclerosis	3.13	1.72			**2.43 ± 0.71**	0.56	0.31			**0.44 ± 0.13**
MCD	8.41	4.63			**6.52 ± 1.89**	1.49	0.82			**1.15 ± 0.34**
FCD I	1.29	0.71			**1.00 ± 0.29**	0.24	0.13			**0.18 ± 0.05**
FCD II	3.74	2.06			**2.90 ± 0.84**	0.65	0.36			**0.51 ± 0.15**
FCD (NOS)	0.68	0.38			**0.53 ± 0.15**	0.13	0.07			**0.1 ± 0.03**
Other MCD	2.72	1.50			**2.11 ± 0.61**	0.49	0.27			**0.38 ± 0.11**
LEAT	5.61	3.09			**4.35 ± 1.26**	1.00	0.55			**0.77 ± 0.23**
Glial scar	1.28	0.70			**0.99 ± 0.29**	0.22	0.13			**0.17 ± 0.05**
Vascular malformation	0.66	0.36			**0.51 ± 0.15**	0.11	0.07			**0.09 ± 0.02**
Encephalitis	0.70	0.38			**0.54 ± 0.16**	0.13	0.07			**0.1 ± 0.03**

Est.1,2,3,4 = Estimate 1,2,3,4; SD = Standard Deviation; MCD = Malformation of cortical development; FCD I = Focal cortical dysplasia type I; FCDII = Focal cortical dysplasia type II; FCD (NOS) = Focal cortical dysplasia (not otherwise specified); LEAT = Low-grade developmental and epilepsy-associated brain tumors.

To evaluate discrepancies between surgical databases, we also calculated additional prevalence and incidence estimates using the two different brain lesion proportions reported by the more recently collected CCF ECOR database (2010–2018) and older EEBB data (1990–2014) (Table S3). The estimates calculated from the older EEBB data alone were similar to those calculated from the combined data (Table 4). However, we observe that, unlike the combined estimates, malformations of cortical development were the most frequent surgically treatable brain lesions observed in the more recent CCF ECOR (2010–2018) for both adults and children (adult population prevalence: 15.58 ± 1.24, pediatric population prevalence: 9.15 ± 3.51).

## Discussion

We estimated both the prevalence and incidence of surgical candidates and the most common surgically amenable epilepsy-associated brain lesions among adults and children in the general population by combining data from the literature with findings from the Cleveland Clinic and a European multicenter cohort. For surgical candidates, we estimate an annual incidence of 2.90 ± 0.85 in 100,000 children and 5.33 ± 0.59 in 100,000 adults as well as an annual period prevalence of 16.36 ± 9.29 in 100,000 children and 44.63 ± 5.63 in 100,000 adults (Fig. 2). From these, we estimate that 30.66% of adults with focal drug-resistant epilepsy and 15.5% of children with drug-resistant epilepsy would qualify as candidates for surgical resection (see Results). Furthermore, we provide the first epidemiological estimates for the most common surgically-treatable epilepsy-associated brain lesions ever reported in the literature for both the adult and pediatric populations (Table 4).

Previous studies estimating the frequency of all individuals with surgically treatable epilepsy have relied on survey-based approaches and clinician estimation to determine the proportion of epilepsy cases amenable to surgical treatment [13], [14]. The results of these studies have varied greatly: from 3% surgical candidates among all epilepsy patients in the United Kingdom [13] to 24% surgical candidates among all epilepsy patients globally [14]. To the best of our knowledge, our study is the first to report surgically amenable epilepsy incidence and prevalence separately for adults and children. Also, unlike previous reports, our surgical candidate estimates are derived solely from empirical data obtained from published reports from a systematic review of the literature.

However, because our estimates are primarily derived from published reports and incorporate data from multiple sources, they are inevitably impacted by publication bias and it is difficult to account for varying underlying population structures. For example, our comprehensive literature review revealed that reports from low-income countries are scarce (Supplementary Appendix S1). As such, most studies included in our analysis either originate from high-income countries (Supplementary Appendix S1) or provide data more easily applied to high-income countries [15]. Accordingly, our surgical candidate and associated brain lesion incidence and prevalence estimates are similarly more easily applied to higher-income countries. However, previous studies have reported that the overall prevalence and incidence of epilepsy is higher in low-middle income countries than in higher-income countries [15], [16] and that these low-resource areas are also those with the largest treatment gap, including epilepsy surgery [17]. Therefore, our reported estimates may potentially be applied to low-middle income countries as conservative lower bound estimates, with the caveat that the true incidence and prevalence of surgically-amenable epilepsies are much likely higher in these countries and the relative distribution of pathologies may be skewed towards epilepsies caused by external factors [15].

Furthermore, our approach accounts for some of the publication bias by generating multiple estimates for both adults and children from different combinations of studies and data points and reporting the average of these (Fig. 2). We also performed multiple sensitivity analyses to evaluate study selection bias and heterogeneity (see Results) which showed that there was no significant difference from the results in our main analysis (Fig. S1 and Table S3). Given the careful analysis and inclusion of a wide range of data, in the absence of empirical data ascertained in a nationwide screen, our results are currently the most robust estimates of candidates for resective epilepsy surgery and associated pathologies.

Understanding the epidemiology of individual types of epilepsy-associated lesions is important to inform and adjust the increasing diversity of surgical treatment modalities, including lesionectomy, temporal lobe resection, hemispherectomy/hemispherotomy, laser ablation, and thermocoagulation [18], [19], [20]. To the best of our knowledge, our study is first to provide population-wide epidemiological estimates for any common surgically remediable brain lesions such as hippocampal sclerosis and focal cortical dysplasia (Table 4). Since our estimates are based on post-surgical histopathological outcomes from resective surgery, we do not provide estimates for other lesional epilepsy-associated pathologies which were not operated on until more recently (e.g., polymicrogyria) or are primarily treated through newer surgical methods such as neuromodulation techniques and laser ablation therapies and therefore only have neuroradiological findings and no histopathological diagnoses (e.g., periventricular nodular heterotopia, deeper brain lesions). However, our study includes incidence and prevalence estimates for the most frequently occurring and commonly resected types of surgically treatable epilepsy-associated brain lesions, based on cohorts from time periods prior to the use of newer surgical methods [2]**.**

Our estimates of lesion incidence and prevalence are based on surgical outcomes from two independent cohorts from different time periods (CCF ECOR: 2010–2018; EEBB: 1990–2014), which reported different proportions for each lesion type (Table 3). Specifically, we observe a lower proportion of hippocampal sclerosis patients and a higher proportion of cortical malformations in the CCF ECOR data compared to the EEBB data (Table 3). Therefore, we provide additional brain lesion incidence and prevalence estimates calculated using the CCF ECOR and EEBB data separately (Table S3). The two datasets may represent different trends in clinical practice and the management of surgical candidates at the Cleveland Clinic Epilepsy Center or other US surgical centers compared to European surgical centers. However, the time period in which these data were collected could potentially also contribute to the observed discrepancy, as the landscape of epilepsy surgery has evolved and seen major changes over the past years. It has previously been reported that clinical practices in epilepsy surgery and the selection of candidates have evolved and seen major changes over the years [18], [21], [22], [23]. Specifically, changing clinical trends describe an increasing proportion of surgical procedures performed for non-temporal lesions compared to temporal lobectomies in recent years [18], [22], [23]. The observed discrepancy between the combined data analysis and the estimates calculated from the more recent CCF ECOR data (a lower proportion of hippocampal sclerosis patients and a higher proportion of cortical malformations) is consistent with these recent reports on changing clinical trends.

Epilepsy surgery has been associated with a reduction in mortality for patients with drug-resistant epilepsy, regardless of whether seizures are completely abolished or their frequency is reduced [24], [25]. Understanding both the frequency of drug-resistant epilepsy patients eligible for resective surgical treatment as well as the frequency of various underlying pathologies is needed to optimize the planning of healthcare services such as the training of specialists, support services provided, and implementation of public health programs. In the absence of wide-scale empirical population-based data, our estimates can help guide patient advocacy groups, clinicians, researchers, and policymakers in community education as well as the development of health care strategies, resource allocation, and reimbursement schedules.

## Funding

This work was supported by National Institutes of Health R01 NS097719, Cleveland Clinic; German Research Council DFG BL421/4-1, University Hospitals Erlangen; European Union, European Reference Networks HP-ERN-2016, grant agreement no. 870280.

## Ethical Statement

We confirm that we have read the Journal's position on issues involved in ethical publication and affirm that this report is consistent with those guidelines. Given the study’s retrospective design, and the fact that only aggregate data and no personal data was utilized, the requirement for informed consent was waived.

## Declaration of Competing Interest

The authors declare that they have no known competing financial interests or personal relationships that could have appeared to influence the work reported in this paper.
